# The joint effect of female sex and food insecurity on self-reported mood disorder among Canadian adults: the Canadian community health survey

**DOI:** 10.1186/s40795-023-00750-9

**Published:** 2023-07-21

**Authors:** James Kotuah Sakeah, Gervin Ane Apatinga, Edgar Balinia Adda, Paschal Awingura Apanga, Carol Vlassoff, Yue Chen

**Affiliations:** 1 School of Epidemiology and Public Health, University of Ottawa, 9 Seguin Street, Gloucester, ON K1J 6P4 Ottawa, Canada; 2 Department of Geography and Planning, University of Saskatchewan, Saskatchewan, Canada; 3 Department of International Development Studies, St Mary’s University, Halifax, Canada; 4 Kentucky Cabinet for Health and Family Services, Kentucky Department for Public Health, Frankfort, USA

**Keywords:** Food insecurity, Mood disorders, General disorders, Sex, Female

## Abstract

**Background:**

Food insecurity is prevalent in Canada and may influence mental health, particularly among females. The present study examined the joint effect of female sex and food insecurity on mood disorders.

**Methods:**

The study used data from 104,420 adults aged 18 years or older who participated in the 2017/2018 Canadian Community Health Survey (CCHS). Log-binomial models explored the independent and joint associations of female sex and food insecurity with the prevalence of self-reported mood disorder. Prevalence ratios (PRs) and 95% confidence intervals (CIs) were estimated. Relative excess risk due to interaction (RERI), attributable proportion (AP), and synergy index (S) were used to assess the additive interaction of female sex and food insecurity. The complex survey design was taken into consideration.

**Results:**

The prevalence of mood disorder was 6.7% for males and 11.4% for females, with an adjusted prevalence ratio being 1.59 (95% CI 1.51, 1.68) for females versus males. Mood disorder was associated with moderate food insecurity (PR 2.06, 95% CI 1.91, 2.23) and severe food insecurity (PR 3.29, 95% CI 3.06, 3.55). There was a significant additive interaction between female sex and food insecurity in association with the prevalence of mood disorders among females aged 18 to 39 years (RERI 1.19, 95% CI 0.27,2.08).

**Conclusion:**

Food insecurity was associated with an increased prevalence of mood disorders, especially in younger females. Interventions that facilitate access to food while being cognizant of the socioeconomic vulnerabilities of females may have substantial benefits for the prevention and management of mood disorders.

## Introduction

Food insecurity is a growing and concerning phenomenon in Canada [1]. Food insecurity, as defined by Health Canada, is “the inability to acquire or consume an adequate diet quality or sufficient quantity of food in socially acceptable ways, or the uncertainty that one will be able to do so“ [2]. Between 2017 and 2018, approximately 12.7% of Canadian households experienced food insecurity [3], and a cross-sectional study from the Canadian perspectives survey series during the COVID-19 pandemic revealed that 14.6% of Canadians live in households facing varying degrees of food insecurity [4]. This situation raises significant concerns as it violates fundamental human rights, undermines empowerment and socioeconomic development, and constrains public health efforts [5, 6].

Numerous studies have established connections between food insecurity and various health and well-being outcomes in high-income countries like Canada. They include physical health problems such as diabetes, malnutrition and cardiovascular diseases [7, 8], as well as socioeconomic challenges such as diminished work productivity and income levels [7]. Additionally, food insecurity has been linked to general mental health conditions such as depression, stress, anxiety, insomnia, suicidal attempts and ideation [9–13]. For instance, Pound and Chen’s (2021) analysis of the CCHS 2015–2016 data showed association between food insecurity and perceived poor or fair mental health among Canadian adults, especially among younger and middle-aged females. However, there remains a significant research gap regarding the examination of prominent and specific mental health outcomes, particularly mood disorders, and their potential variations among subpopulations using nationally representative data. In Canada, mood disorders are among the most prevalent mental illnesses, with an estimated 11.6% of the population aged 18 years or older reporting these disorders, and an additional 27% indicating that the disorders have affected their lives [14, 15]. These disorders have consistently exhibited a higher prevalence among females in Canada. Recent research has further underscored the disproportionate impact of these outcomes on females compared to males, partially attributed to the connection between food security and financial ability, with females overrepresented in low-income groups [1, 12, 13, 16, 17].

To address this research gap, the present study examined the additive interaction between female sex and food insecurity in relation to self-reported mood disorder among adults using data from the Canadian Community Health Survey (CCHS) and a methodology applied elsewhere [13, 18]. We hypothesized that the combination of female sex and food insecurity will have a synergistic effect on self-reported mood disorder. This study will provide a nuanced understanding of the relationship between food insecurity and self-reported mood disorders, with the goal of identifying individuals who may benefit more from interventions that improve access to food resources and focus on the prevention, early detection, and management of mood disorders.

## Methods

### Design

This cross-sectional study utilized secondary data from the 2017–2018 Canadian Community Health Survey [18] public use microdata file and examined the joint effect of female sex and food insecurity on self-reported mood disorder [18]. It follows the guidelines of Strengthening of the Reporting of Observational Studies in Epidemiology (STROBE) [19] and Reporting of Studies Conducted Using Observational Routinely Collected Health Data (RECORD) [20]. The CCHS is a nationally representative survey that uses a complex survey design to sample the participants [21].

### Study population and variables

The CCHS survey collects data on health status, utilization of the health system, and health determinants of the Canadian population [18]. Data are collected on people aged 12 years and older in all provinces and territories but excludes residents of Indian reserves, healthcare institutions, some remote areas and full-time members of the Canadian armed forces [18]. The current analysis used data from 104,420 Canadian adults aged 18 years or above who participated in the 2017–2018 CCHS. We excluded participants who were under 18 years of age (*n* = 8,621) or who did not respond to the question on mood disorders (*n* = 249).

The outcome variable was self-reported mood disorder. This was dichotomized based on a “Yes” or “No” response to the question, “Do you have a mood disorder such as depression, bipolar disorder, mania or dysthymia?”.

Our exposures of interest were food security and sex. Food insecurity was based on a set of 10 questions that described the food security of a household’s adult members in the previous 12 months. Study participants were grouped into three categories: [1] food secure (none or one affirmative response indicating difficulty with income-related food access); [2] moderately food insecure (two to five affirmative responses of indicating compromise in the quality and/or quantity of food consumed); and [3] severely food insecure (six or more affirmative responses indicating compromise in the quality and/or quantity of food consumed) [18]. Sex was self-reported as being male or female.

Covariates were those identified in the literature as statistically associated with mental health [1, 5–10] and food security status [1–6], as available in the CCHS dataset. These included age (18–29, 30–39, 40–49, 50–59, 60–69, 70–79, 80 + years), annual household income (<$40,000, $40,000–79,999, ≥ $80, 000), marital status (single, married, divorced), education level (less than secondary school graduation, secondary school graduation, post-secondary education), race (white, non-white), and social provisions (having, not having a positive and meaningful type of support, a composite measure derived from five sub-scales that include guidance, integration, attachment, reassurance of worth, and reliable alliance).

### Statistical analysis

We accounted for the complex survey design of the CCHS by applying sampling weights and average design effect for point and variance estimates. The chi-square significance test was used to examine associations between our variables of interest and self-reported mood disorders, and a *p*-value of < 0.05 determined statistical significance.

We used a log-binomial regression model to examine sex and food insecurity in association with the prevalence of self-reported mood disorder. We also assessed if this relationship was modified by age. Age was assessed as an effect modifier with a *p*-value of < 0.05 considered as being statistically significant; hence age-specific analyses were conducted in younger adults (18–39 years), middle-aged adults (40–59 years) and older adults (60 + years). Prevalence ratios were generated with their 95% confidence intervals (CIs). Model parameters were estimated using maximum likelihood estimation.

We assessed the interaction of sex and food insecurity in association with self-reported mood disorders on the additive scale. The additive scale was chosen because it reliably identifies the target individuals to intervene to achieve a significant public health impact [22, 23]. The assessment of additive interaction was done using the relative excess risk due to interaction (RERI), attributable portion due to interaction (AP) and synergy index (S) and their respective 95% CIs measures [24]. The determination of the presence of additive interaction was based on whether the RERI and AP were significantly different from 0 and the synergy index was significantly different from 1. Statistical analyses were performed using SAS 9.4 software (SAS Institute).

## Results

**Fig. 1:**
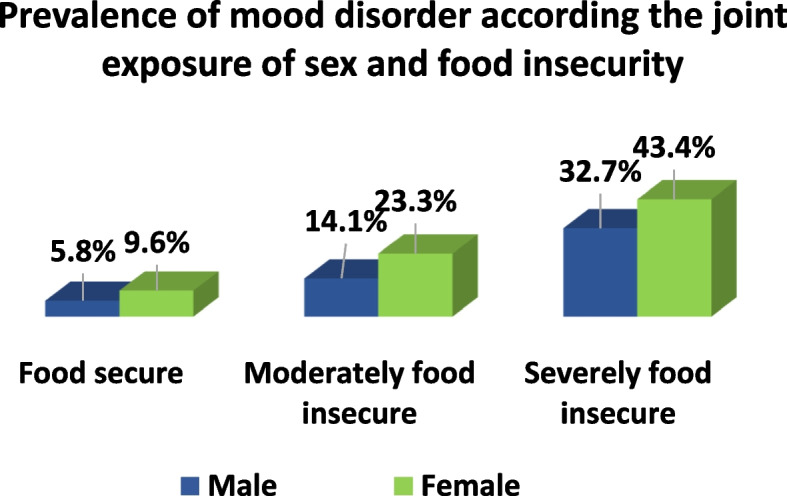
Prevalence of self-reported mood disorder according to joint exposure of sex and food security in Canadian adults

Table 1 presents the prevalence of self-reported mood disorders across various categories of our variables of interest. All the study variables were significantly associated with the prevalence of self-reported mood disorder (*p* < 0. 0001). The prevalence of self-reported mood disorders was 4.7% higher among females than males. Individuals with severe food insecurity had a higher prevalence of self-reported mood disorder (38.7%) compared to people who were moderately food-insecure (19.8%), and those who were food-secure (7.7%). When we examined the prevalence of self-reported mood disorders among the joint exposure of sex and food security, females who were severely food insecure had the highest prevalence of self-reported mood disorder (43.4%) (Fig. 1).

**Table 1:** Prevalence of self-reported mood disorder by sex and other risk factors in the Canadian population

Variable	No.	Cases of Mood disorders	%^a^	*P*-value
**Sex**				
Male	47,856	3642	6.7	< 0.0001
Female	56,564	6980	11.4	
**Food security**				
Food secure	95,399	8027	7.7	< 0.0001
Moderately food insecure	5650	1240	19.8	
Severely food insecure	3371	1355	38.7	
**Age (years)**				
18–29	13,948	1618	10.0	< 0.0001
30–39	15,710	1675	8.9	
40–49	14,302	1653	9.6	
50–59	1798	2250	10.3	
60–69	20,867	2078	9.1	
70–79	14,095	979	6.3	
80+	7510	369	5.7	
**Marital Status**				
Single	24,089	3401	12.4	< 0.0001
Married	57,318	4355	7.1	
Divorced	23,013	2866	12.4	
**Family income**				
<$40, 000	27,893	4292	14.2	< 0.0001
$40,000–79,000	31,114	2952	9.2	
≥ $80,000	45,413	3378	7.1	
**Race**				
Non-White	12,090	707	5.1	< 0.0001
White	92,330	9915	10.2	
**Highest level of education**				
< Secondary school	15,963	1749	10.6	< 0.0001
Secondary school completed	24,635	2854	10.6	
>Secondary school	63,822	6019	8.3	
**Having social provisions**				
No	73,035	7204	8.8	< 0.0001
Yes	31,385	3418	10.0	

^a^Weighted to the Canadian population

Table 2 shows the prevalence ratios for mood disorder in relation to sex and food security status from the log-binomial regression analysis. In the adjusted model for the effects of female sex and food insecurity, females had a significantly higher prevalence of self-reported mood disorder than males (PR 1.59, 95% CI 1.51, 1.68). The prevalence of mood disorder was also significantly higher for those with moderate food insecurity (PR 2.06, 95% CI 1.91, 2.23) and severe food insecurity (PR 3.29, 95% CI 3.06, 3.55) compared with food secure individuals. In the adjusted model for the joint effect of female sex and food insecurity, the overall prevalence ratio for self-reported mood disorder for females experiencing severe food insecurity was 5.12 (95% CI 4.66, 5.61) compared with food-secure men. Relative to men with food security in each age group, the adjusted prevalence ratios for self-reported mood disorder for females experiencing severe food insecurity were 5.97 (95% CI 5.21, 6.79) for younger adults (18–39 years), 4.78 (95% CI 4.17, 5.44) for middle-aged adults (40–59 years), and 4.23 (95% CI 2.86, 5.86) for older adults (60 + years).

**Table 2:** Unadjusted and adjusted prevalence ratios (PRs) and 95% confidence intervals (CIs) for the associations of sex and food security status with self-reported mood disorder

	Model 1 Unadjusted^a^				Model 2 Adjusted^b^
Variable	PR (95% CI)				PR (95% CI)
**Sex**					
Male	Reference			Reference	
Female	1.61	(1.53, 1.70)		1.59	(1.51, 1.68)
**Food security**					
Food secure	Reference			Reference	
Moderately food insecure	2.44	(2.26, 2.62)		2.06	(1.91, 2.23)
Severely food insecure	4.84	(4.51, 5.17)		3.29	(3.06, 3.55)
**Joint exposure of sex and food security**					
** Overall**					
** Male**					
Food secure	Reference			Reference	
Moderately food insecure	2.42	(2.09, 2.78)		2.12	(1.84, 2.42)
Severely food insecure	5.66	(5.01, 6.35)		3.82	(3.37, 4.29)
** Female**					
Food secure	1.66	(1.57, 1.76)		1.65	(1.55, 1.75)
Moderately food insecure	4.05	(3.67, 4.44)		3.35	(3.04, 3.69)
Severely food insecure	7.52	(6.86, 8.21)		5.12	(4.66, 5.61)
** Ages 18 to 39**					
Male					
Food secure				Reference	
Moderately food insecure	2.54	(2.05, 3.09)		2.33	(1.88, 2.84)
Severely food insecure	5.41	(4.44, 6.49)		3.90	(3.20, 4.68)
** Female**					
Food secure	1.81	(1.64, 2.00)		1.88	(1.70, 2.08)
Moderately food insecure	4.22	(3.65, 4.85)		3.74	(3.24, 4.30)
Severely food insecure	8.20	(7.15, 9.36)		5.97	(5.21, 6.79)
**Age 40 to 59 years**					
** Male**					
Food secure				Reference	
Moderately food insecure	2.54	(1.88, 2.84)		2.24	(1.82, 2.72)
Severely food insecure	5.91	(5.01, 6.89)		3.96	(3.36, 4.61)
** Female**					
Food secure	1.70	(1.56, 1.86)		1.66	(1.52, 1.81)
Moderately food insecure	4.01	(3.47, 4.60)		3.29	(2.85, 3.77)
Severely food insecure	7.21	(6.29, 8.20)		4.78	(4.17, 5.44)
**Ages 60+**					
** Male**					
Food secure				Reference	
Moderately food insecure	1.10	(0.48, 2.09)		1.02	(0.45, 1.92)
Severely food insecure	4.32	(2.18, 7.19)		3.55	(1.81, 5.80)
** Female**					
Food secure	1.37	(1.20, 1.58)		1.21	(1.06, 1.40)
Moderately food insecure	3.37	(2.36, 4.57)		2.59	(1.83, 3.53)
Severely food insecure	5.38	(3.62, 7.50)		4.23	(2.86, 5.86)

Definitions: *PR *Prevalence ratio, *CI *Confidence interval

^a^Model 1 variables: Main exposures only (sex and food security status, or the combination sex and food security status)

^b^Model 2 variables: Main exposures (sex and food security status or the combination of sex and food security), age, marital status, race, education, and household income

Table 3 shows the additive interaction between female sex and food insecurity measured by RERI, AP, and S index [24]. The results show a significant synergism between female sex and severe food insecurity on the prevalence of self-reported mood disorder. The synergistic effect was still present but only in the younger adults (18–39 years) when stratified by age (RERI 1.19, 95% CI 0.29, 2.08; AP 0.20, 95% CI 0.06, 0.34; S 1.31, 95% 1.06, 1.63).

**Table 3:** The joint effect of female sex and severe food insecurity on self-reported mood disorders overall and stratified by age

	Additive interaction (95% CI)		
Exposure	RERI	AP	S index
**Overall**			
Female v. male sex with moderate food insecurity	**0.58 (0.05, 1.10)**	**0.17 (0.03, 0.31)**	**1.32 (1.03, 1.70)**
Female v. male sex with severe food insecurity	**0.66 (0.11, 1.27)**	**0.13 (0.03, 0.23)**	**1.19 (1.03, 1.38)**
**Ages 18 to 39 years**			
Female v. male sex with moderate food insecurity	0.53 (-0.09, 1.16)	0.14 (-0.02, 0.30)	1.24 (0.96, 1.61)
** Female v. male sex with severe food insecurity**	**1.19 (0.29, 2.08)**	**0.20 (0.06, 0.34)**	**1.31 (1.06, 1.63)**
**Ages 40 to 49 years**			
Female v. male sex with moderate food insecurity	0.38 (-0.20, 0.97)	0.12 (-0.05, 0.29)	1.20 (0.90, 1.60)
Female v. male sex with severe food insecurity	0.16 (-0.59, 0.91)	0.03 (-0.12, 0.19)	1.04 (0.85, 1.28)
**Ages 60 + years**			
Female v. male sex with moderate food insecurity	1.35 (0.27, 2.43)	0.52 (0.20, 0.84)	6.64 (0.27, 163.69)
Female v. male sex with severe food insecurity	0.47 (-1.99, 2.92)	0.11 (-0.45, 0.67)	1.17 (0.50, 2.73)

*Abbreviations:* *AP *Attributable portion due to interaction, *CI *Confidence interval,

*RERI *Relative excess risk due to interaction

## Discussion

In this study, adults with food insecurity, moderate or severe, had a significantly higher prevalence of self-reported mood disorder compared to those who were food secure. Self-reported mood disorder, defined as individuals who reported having depression, bipolar, mania, or dysthymia, was more prevalent in females (18.1%) than in males (11.4%). There was a synergism between female sex and food insecurity in relation to self-reported mood disorder. Food insecurity may influence the psychological functioning of adults, and food insecurity is a significant stressor, preeminent in the worry and anxiety usually associated with it [1, 10, 13, 25–27]. There is evidence that limited food accessibility is associated with a rise in cortisol levels. High cortisol levels factor in chronic psychosocial distress, which alters mood, cognition, and behaviour [26–30].

Conversely, individuals with severe food insecurity may be at a higher risk of inadequate consumption of essential nutrients that can substantially impact mental health, as seen in previous studies [31]. Another plausible reason that females are more likely to self-report mood disorders due to food insecurity could be income levels, as income is a strong determinant of food security [1, 17, 32], and females are disproportionately represented in low-income groups [1, 12, 13, 16, 17]. Income provides purchasing power, and lack of it can be a considerable stressor [1], especially for females whose gender roles prescribe them to be the primary providers of food and nourishment in the family. Prolonged unavailability of income can result in chronic stress, further contributing to poor mental health outcomes [1, 17, 32]. Alternatively, mood disorders could hinder females from effectively participating in the labour market, exacerbating their vulnerability to food insecurity [1, 33] and thus rendering them into a vicious cycle of poverty and mental health vulnerabilities.

The strong dose-response association between food insecurity and self-reported mood disorder in our study is consistent with the results of prior studies [13, 25, 34, 35]. However, given the cross-sectional design of this study, a causal relationship between food insecurity and self-reported mood disorder cannot be ascertained.

Our findings also showed a synergism between female sex and severe food insecurity, although this was only statistically significant among young females. This observation aligns with previous studies [1, 12, 13, 16, 35], notably the study conducted by Pound and Chen (2021) using a similar methodology and the 2015–2016 CCHS dataset. They identified an additive interaction between female sex and food insecurity, with a higher risk observed among middle-aged females. These findings suggest that females are at greater risk for mood disorders concerning food insecurity. Plausible reasons why young adult females are at higher risk are not entirely clear. Some studies have suggested that early adulthood transitions such as independence, employment, new relationships, and motherhood can take a toll on the psychological functioning of young females [36]. In contrast, middle-aged and older females tend to exhibit greater financial stability due to established careers, accumulated savings, social safety net programs and larger social support networks [37–40].

In Canada, wage differentials between males and females [41] may also be a factor, as females tend to have lower pay and to work in part-time jobs [42] to accommodate their domestic and child-raising chores [43], all of which may add to their mental stress. Such factors, combined with stressful situations like food insecurity, may have an adverse impact on mental health. Hence, given that access to food can be modified, interventions targeted at this group, food assistance programs [44], social support, and income and employment incentives may have substantial benefits for the prevention and management of mood disorders. Evidence from the literature shows that food assistance programs have the potential to reduce households’ food-related hardships and offsets emotional and psychological distress [45–47]. More research is needed to investigate the different mood responses of males and females to food insecurity and the possible interventions to address them.

### Limitations

The present study has some limitations. First, given that mood disorder and food security status were self-reported, there could be a recall and reporting bias. However, we expect recall and reporting bias to be similar among participants who reported mood disorders or not. Secondly, the CCHS data collection tool provides an indicator of the quantitative food security situations and economic access to food. Therefore, these measures are likely to miss relevant mental health components such as the social desirability of food acquisition strategies [1, 48]. For example, food banks may help alleviate food insecurity, but people may avoid using them because of the stigma attached to accessing food there, or food may be inappropriate for specific religious or cultural groups or those with special dietary needs. Not measuring these factors may have resulted in our underestimating the impact of food insecurity on mood disorder. Thirdly, the cross-sectional nature of this study does not allow for establishing the causal relationship between the joint exposure of female sex and food insecurity and mood disorder. This study might also be subjected to selection bias as the CCHS excludes data on residents of Indian reserves, healthcare institutions and full-time members of the Canadian armed. Additionally, the findings cannot be fully explained based on the available data due to the lack of specific information on reasons for food insecurity among adults or categorization by factors such as sex or age. Finally, it is important to note that the CCHS data used in this study predates the COVID-19 pandemic. Therefore, caution should be exercised when generalizing the obtained results to the current general population. Notwithstanding its limitations, the use of a large sample size, along with the application of bootstrap weights from the CCHS data, is a definite strength of this study and also makes the results nationally representative.

## Conclusion

Our study findings provide evidence for a significant synergistic effect between female sex and severe food insecurity, leading to a higher prevalence of self-reported mood disorders, especially in young adults. These results underscore the particular vulnerability of young females to mood disorders associated with food insecurity. To effectively address this issue, it is essential to develop comprehensive interventions that address both mental health and food needs, with careful consideration of the distinct challenges faced by various age groups, particularly for females.

## Data Availability

Public Use Data (Link: https://www150.statcan.gc.ca/n1/en/catalogue/82M0013X2020001).

## Code Availability

Yes.
